# A Case Report of the Reconstruction of a Bone Defect Following Resection of a Comminuted Fracture of the Lateral Clavicle Using a Titanium Prosthesis

**DOI:** 10.3389/fsurg.2021.646989

**Published:** 2021-09-03

**Authors:** Sahar Ahmed Abdalbary, Sherif M. Amr, Khaled Abdelghany, Amr A. Nssef, Ehab A. A. El-Shaarawy

**Affiliations:** ^1^Department of Orthopaedic Physical Therapy, Faculty of Physical Therapy, Nahda University, Beni Suef, Egypt; ^2^Department of Orthopaedic Surgery, Faculty of Medicine, Cairo University, Giza, Egypt; ^3^Advanced Manufacturing Division, The Central Metallurgical Research and Development Institute, Helwan, Egypt; ^4^Department of Intervention Radiology, Radiology and Vascular Imaging, Faculty of Medicine, Cairo University, Giza, Egypt; ^5^Department of Anatomy and Embryology, Faculty of Medicine, Cairo University, Giza, Egypt

**Keywords:** finite element analysis, comminuted fracture, clavicle, fracture, clavicle prosthesis

## Abstract

**Introduction:** This case report describes the reconstruction of a severe comminuted fracture and bone defect in the lateral half of the clavicle using a novel titanium prosthesis. This unique prosthesis has been specifically designed and three dimensionally printed for the clavicle, as opposed to the Oklahoma cemented composite prosthesis used in common practice. The aims of this study were to: (1) describe the prosthesis, its stress analysis, and its surgical fixation and (2) to demonstrate the results of the 2-year follow-up of the patient with the lateral clavicle prosthesis.

**Patient's Main Concerns:** A 20-year-old, right-handed woman complaining of severe pain in the right shoulder was admitted to our hospital following a traffic accident. Physical examination revealed pain, swelling, tenderness, limb weakness, asymmetric posturing, and loss of function in the right shoulder.

**Diagnosis, Intervention, and Outcomes:** Radiographic evaluation in the emergency room showed complete destruction with a comminuted fracture of the lateral half of the right clavicle and a comminuted fracture of the coracoid. We designed a new prosthesis for the lateral half of the clavicle, which was then tested by finite element analysis and implanted. Use of the new prosthesis was effective in the reconstruction of the comminuted fracture in the lateral half of the clavicle. After 2 years of follow-up, the patient had an aesthetically acceptable curve and was able to perform her activities of daily living. Her pain was relieved, and the disabilities of the arm, shoulder, and hand score improved. Active range of motion of the shoulder joint and muscle strength were also improved.

**Conclusion:** This novel prosthesis is recommended for reconstruction of the lateral half of the clavicle following development of bony defects due to fracture. Our patient achieved functional and aesthetic satisfaction with this prosthesis.

## Introduction

The clavicle, acting as a strut between the scapula and the sternum, articulates with the sternal manubrium medially and with the acromion of the scapula laterally (1). The clavicle contributes to the strength, coordinated scapulohumeral rhythm, and overall range of motion (ROM) of the shoulder girdle (2).

Fractures of the clavicle are common in adults and represent about 4% of all fractures. Further, 21% of clavicle fractures affect the lateral half (3). Clavicular fractures are caused mostly from direct injury, accompanied by a fall on the point of the shoulder, which is the most clinical and biomechanical mechanism of fracture (4).

Recently, to decrease the high rate of surgical complications after claviculectomy, some surgeons suggest a new method for reconstruction of bony defects with autogenous bone or allografts bone to protect the subclavian vessels and brachial plexus, restore the shape of the shoulder, and decrease pain (5). Outcomes of midshaft clavicular malunion, including restoration of length and alignment, soft-tissue preservation, use of local bone graft, and plate fixation, is a reliable treatment option, regardless of the time since fracture (6). Vartanian et al. (7), report the use of an Oklahoma prosthesis, which is a cemented composite prosthesis used to reconstruct bony defects after metastatic tumor resection of the medial third of the clavicle.

Computer-aided engineering technology has many applications in the medical field to generate a surface model of the prosthesis mirrored from the contralateral healthy side (8). Finite element analysis is used to examine the biomechanics of the clavicle reconstruction plate, which are calculated by the quantity of the forces applied by the muscles as a result of the surgical technique (9).

We present the case of a severe comminuted bony defect following fracture in the lateral half of the clavicle treated using a titanium prosthesis. To resolve the bony defect, we designed a novel titanium prosthesis for the lateral half of the clavicle.

In this study, we designed the titanium prosthesis and tested it by finite element stress analysis and inserted it to fill the defect at the site of bone loss in the clavicle. The aims of this study were to: (1) describe the prosthesis, its stress analysis, and its surgical fixation and (2) to demonstrate the results of the 2-year follow-up of the patient with the lateral clavicle prosthesis. To our knowledge, the presentation, findings, and management of this case have not been previously described in the literature.

## Case Report

A 20-year-old, right-handed woman complaining of severe pain in the right shoulder was admitted to our hospital following a traffic accident. Physical examination revealed pain, swelling, tenderness, limb weakness, asymmetric posturing, and loss of function in the right shoulder.

**Figure 1:**
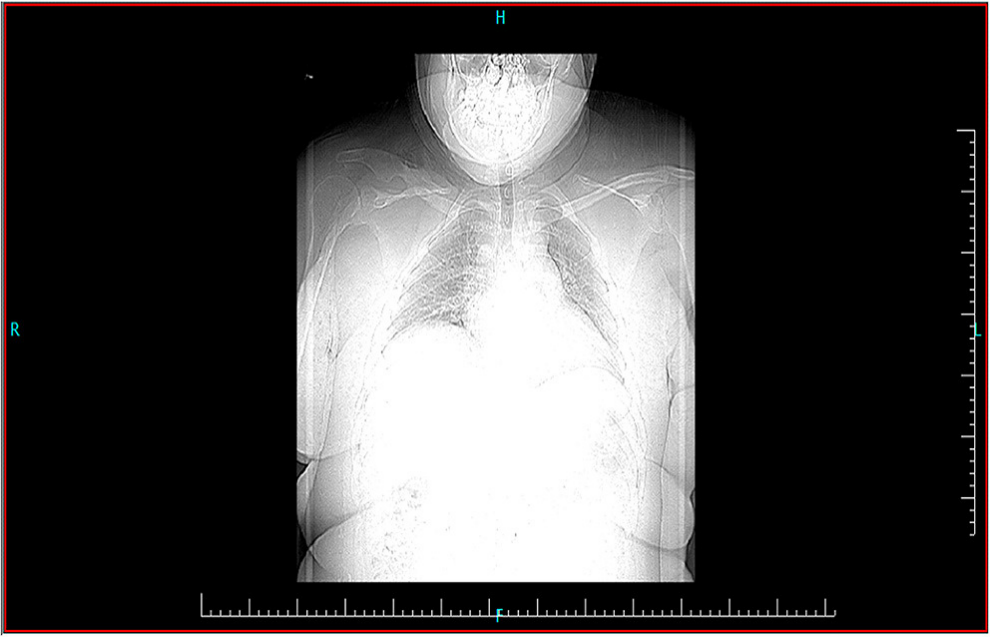
Preoperative computed tomography scan revealing the scope of the lesion. This was essential in identifying the small bone fragments separated from the fracture.

Radiographic evaluation in the emergency room showed complete destruction with a comminuted fracture of the lateral half of the right clavicle and a comminuted fracture of the coracoid. A computed tomography (CT) scan revealed the scope of the lesion and was essential in identifying the small bone fragments separated from the fracture (Figure 1).

A preoperative assessment was performed for pain using the visual analog scale (VAS) score with a result of 9. The preoperative disabilities of the arm, shoulder, and hand (DASH) score (10) was 98.3. A higher score means greater disability, with 100 points indicating a complete disability of the extremity and 0 points indicating a perfect extremity.

The patient was informed that data concerning her status would be submitted for publication and the patient agreed and signed the form providing written informed consent to participate in the study. The study was registered on ClinicalTrials.gov (NCT03577678).

### Manufacturing of Prosthesis and Finite Element Analysis

**Figure 2:**
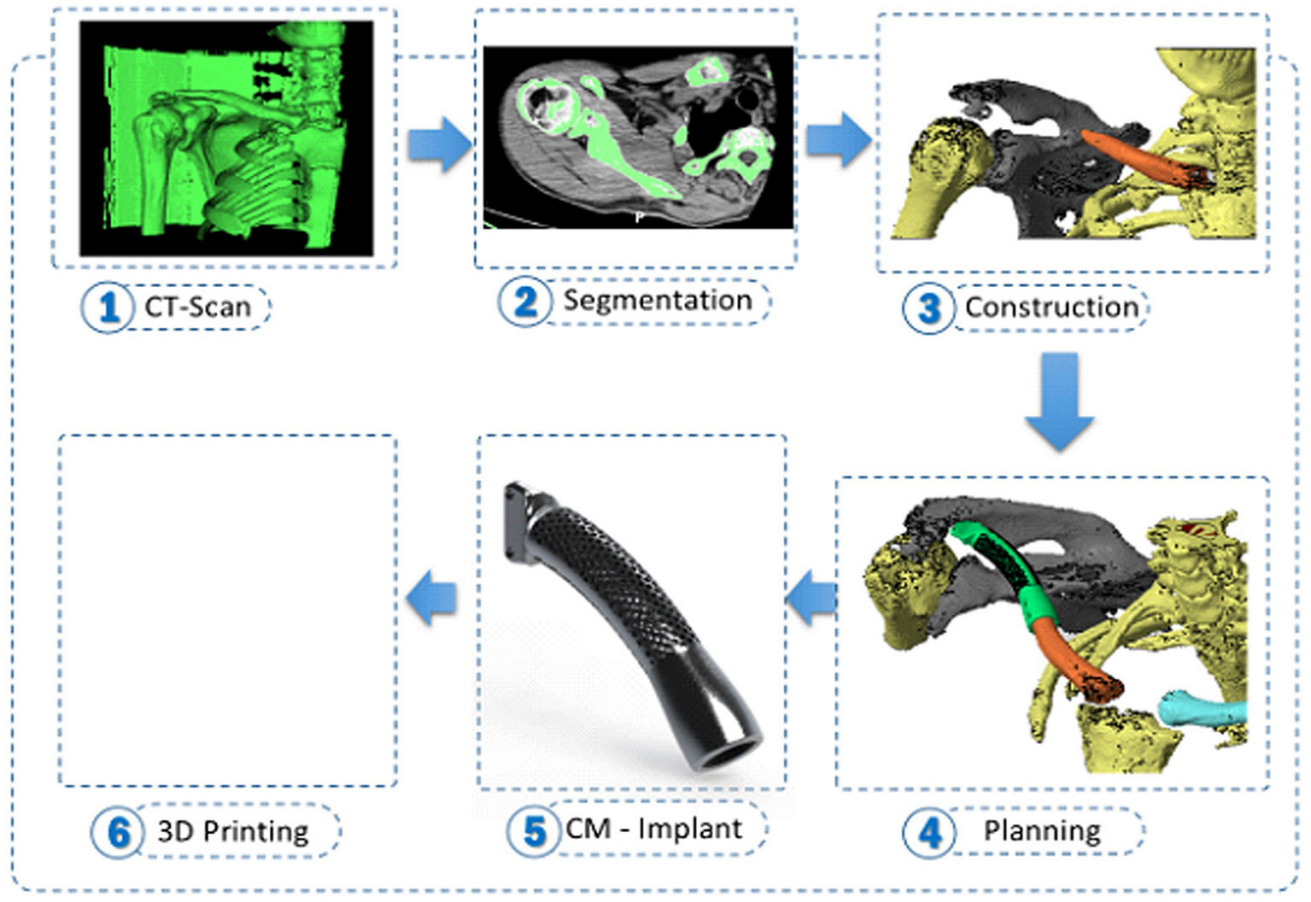
The process of manufacturing of the prosthesis. The three-dimensional model of the clavicle was developed using the digital imaging and communication in medicine format, and image segmentation was performed using the Mimics software (Materialize NV).

**Figure 3:**
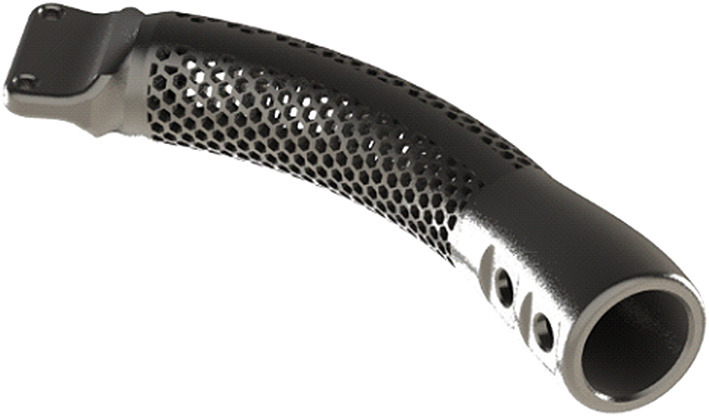
Titanium design of the lateral half of the clavicle. The size of the prosthesis corresponded to that of the lost portion of the clavicle, and the prosthesis had a porous structure to reduce the modulus.

A normal clavicle three-dimensional (3D) geometry model was designed using data extracted from the CT scan. The 3D model of the clavicle was developed using the digital imaging and communication in medicine format, and image segmentation was performed using the Mimics software (Materialize NV) (Figure 2). The size of the prosthesis corresponded to that of the lost portion of the clavicle,

The Prosthesis was manufactured by using 3D printer selective laser melting. The material used was Ti-6Al-4V alloy powder. The prosthesis Size of pores were 900 microns air and 100 microns solid, and the Volume of pores to the e whole implant was 60% while Weight of pores to whole implant body is 38%. The prosthesis was structured from mesh and 2 holes on the medial part of both sides to reduce the modulus. The surface of the prosthesis was polished without any coating (Figure 3). We created three designs to reach the best one which simulate bony part of clavicle.

**Figure 4:**
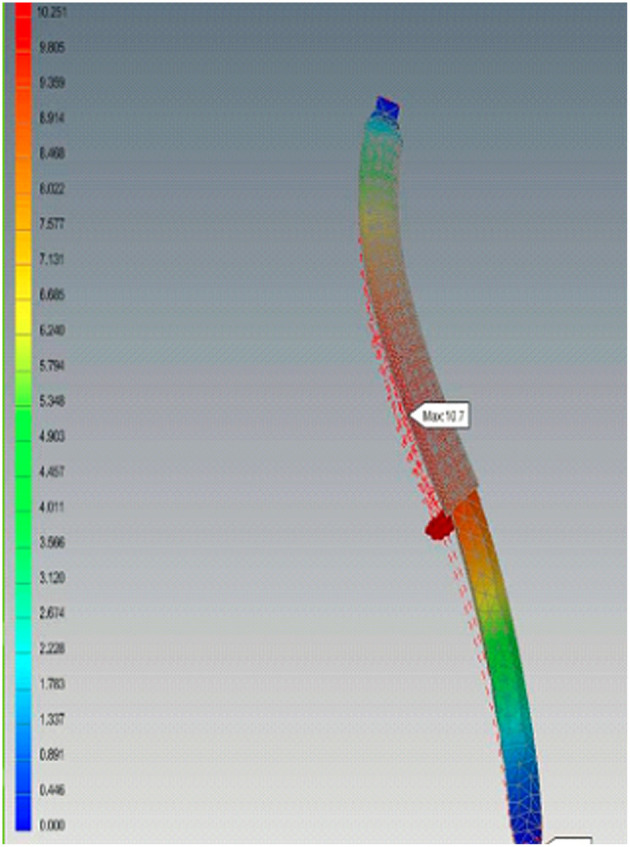
Finite element analysis. A comparison of the stresses predicted and the load location suggest that the results could be in the same range.

After completion, the 3D computer models and the designed model were imported into the ANSYS Workbench 15.0 (ANSYS Inc., Canonsburg, PA, USA) for finite element analysis. The stress distribution and deformation were calculated using the ANSYS Workbench. The boundary conditions applied are two fixed points at the ends of the implant on the lateral side and medial side. Thereafter, the effect of dynamic load location on the clavicle is applied with tension loads in two directions of the later side and medial side with an amount assumed to be 150 N to simulate the real case. The maximum stress was tested. The maximum stress was present at the middle third of the clavicle length, which is a characteristic of clavicle fractures in real life. While the maximum elongation was 50 microns and 75 microns in the lateral and medial sides, respectively. Thus, a comparison of the stresses predicted and the load location by finite element analysis suggested that the results could be in the same range (Figure 4).

We validated the results of FEA to check the results given by FEA software. This was done by comparison with experimental data, and comparison with other similar computation techniques.

### Surgical Technique

The patient underwent the operation in the supine position. The operative site was sterilized from the lateral border of the acromion to the sternum, and then a horizontal skin incision was made on the superior surface of the clavicle. The skin, platysma, and subcutaneous tissue were raised, with care taken to avoid injury to the supraclavicular nerves. The fracture site was exposed and inspected; thereafter, the small fragments and sharp fracture ends were removed.

**Figure 5:**
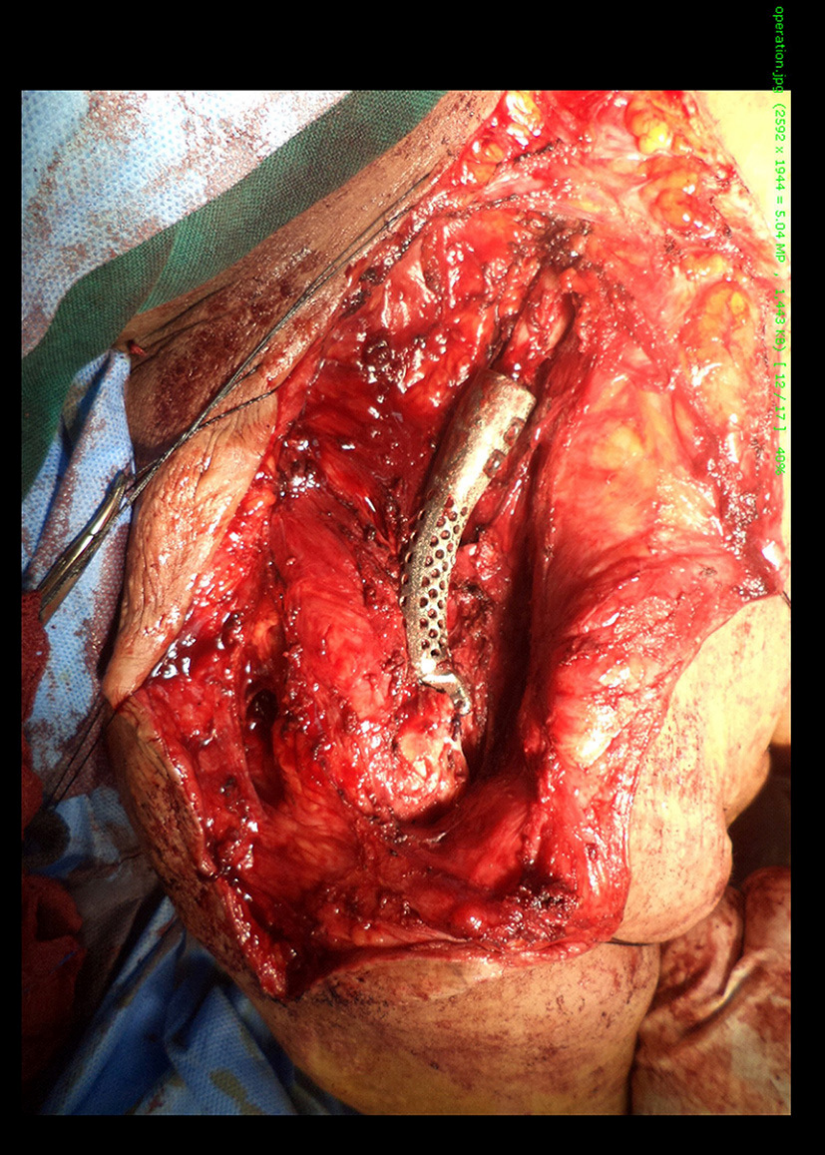
Implantation of the prosthesis. The prosthesis was fixed with the remaining normal portion of the clavicle using a press fit, and the entire prosthesis was filled with a synthetic bone substitute.

The prosthesis was fixed with the remaining normal portion of the clavicle using a press fit, and the entire prosthesis was filled with a synthetic bone substitute (Figure 5). The prosthesis was implanted and fixed to the acromion with non-absorbable sutures through small holes on the surface of the prosthesis. Thereafter, the wound was closed. The operating time was 1.5 h, the blood loss was 1 L, and there were no intra-operative complications.

### Postoperative Care

The arm was maintained in a sling throughout the day for 2 weeks. Thereafter, active assisted ROM exercises of the shoulder at the scapular plane were initiated. Full active motion was started at 4 weeks, and strengthening and resistive exercises of the shoulder girdle were started at 6 weeks up to 12 weeks. The progress of the exercises was dependent on the tolerance of the patient. By 6 months, the patient resumed her normal activities of daily living.

### Outcomes

**Figure 6:**
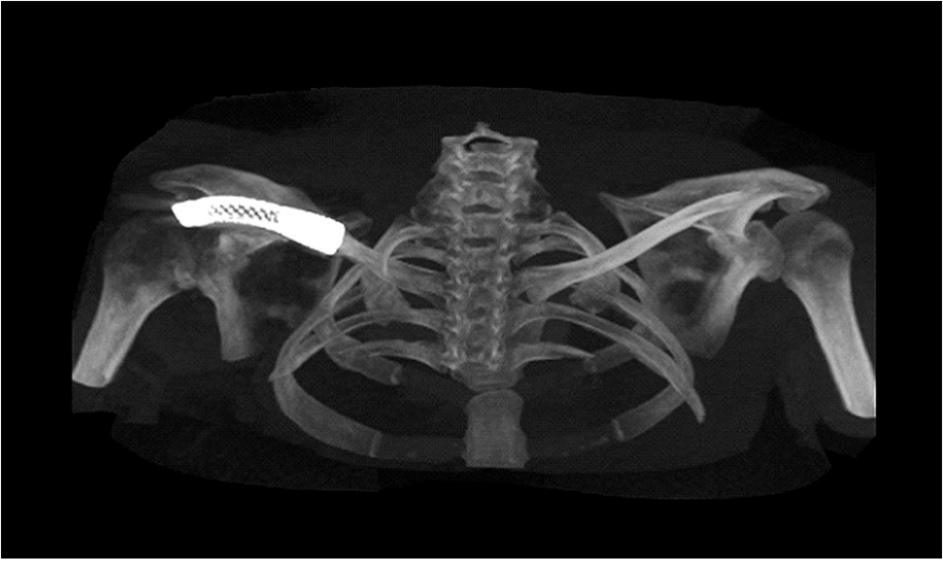
Postoperative computed tomography scan 6 months after the operation revealed a good position for the prosthesis and no evidence of a stress fracture.

The VAS score for pain was 2, while the DASH score was 28, after 2 years post operation. The CT scan 6 months after the operation (Figure 6) revealed a good position for the prosthesis and no evidence of a stress fracture. Active ROM results of the shoulder at 1-year follow-up were flexion 125°, abduction 110°, external rotation 50°, and internal rotation 70°.

**Figure 7:**
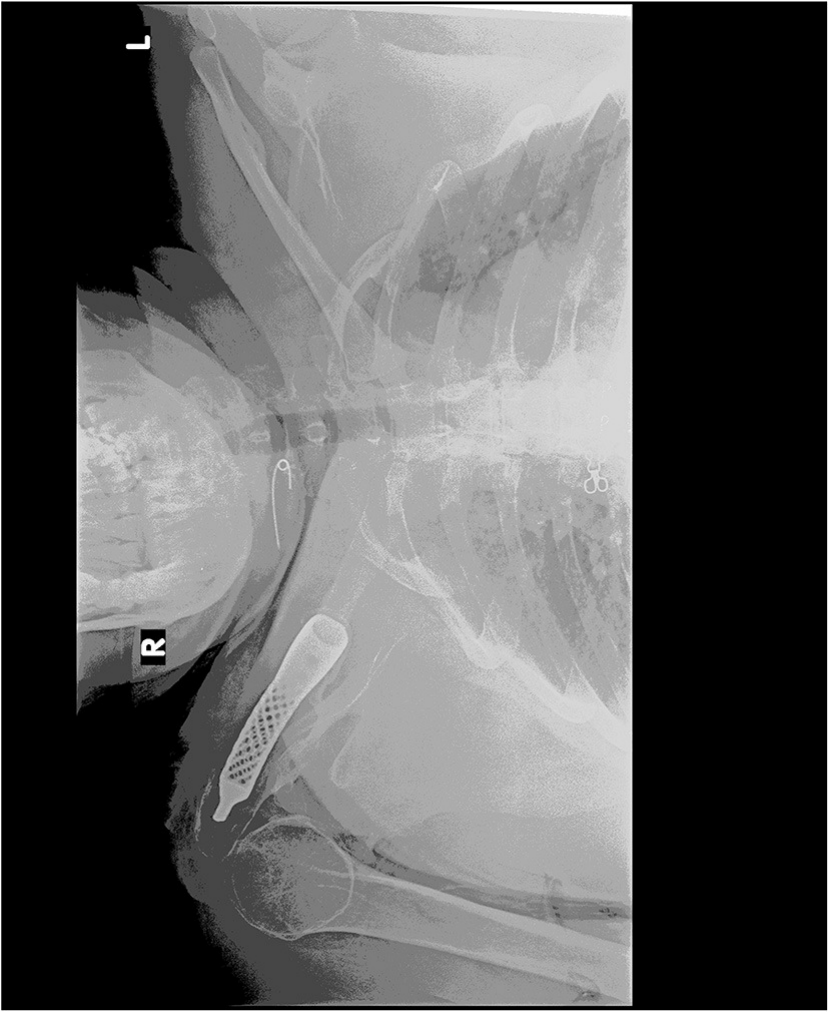
Postoperative radiograph at 2-year follow-up revealed a good position of the prosthesis.

A radiographic examination at 2-year follow-up (Figure 7) revealed a good position of the prosthesis and no bony changes found. Muscle strengths compared with the uninjured shoulder at 2-year follow-up were maximum flexion strength 73%, maximum abduction strength 71%, maximum external rotation strength 65%, and maximum internal rotation strength 77%. Our patient was able to perform her activities of daily living but was not able to participate in sports activities that required a wider shoulder ROM, such as throwing.

## Discussion

This study described a case with comminuted lateral half clavicle fracture that was reconstructed using a 3D printed prosthesis for treatment of the bone defect. The postoperative contours of the shoulder were acceptable to the patient, and there was no evidence of related complications, like infection or a rejection reaction. The procedure maintained the supportive function of the shoulder and protection for the major vessels and nerves at the base of neck.

The clavicle provides protection for the major vessels and nerves at the base of the neck and maintains an aesthetic function by defining graceful curves. It also works as a support to hold the glenohumeral joint and increase the ROM (11). To our knowledge, there are two studies regarding bone defect reconstruction with cement prosthesis after clavicle tumor resections (5, 7), and 1 about a titanium prosthesis after total claviculectomy (11). The use of customized 3D printing prostheses has proved beneficial in many surgeries (12). In this study, 3D printing was used to construct a lateral half model of the clavicle, and the geometrical borders of the contralateral healthy clavicle were used to design the prosthesis. The largest differences between the reconstructed portion and the normal one were seen at the insertion sites of the tendons and ligaments, because the action of the muscles was limited in the reconstruction of the lateral half of the clavicle (13).

The Ti-6Al-4V alloy is considered biocompatible to the human body. One of the main desirable properties of biomaterials for orthopedic implants is the low modulus of elasticity due to bone reabsorption, besides biocompatibility, corrosion resistance, mechanical strength, fatigue resistance and wear resistance, which is especially the case of metallic materials.

The surface of prosthesis has a direct influence on its anchorage in bone. It is responsible for direct contact with patient tissues and is a key factor for Osseo integration (14); we designed the prosthesis from Ti-6Al-4V alloy.

Selective laser melting is one of the most widely used additive manufacturing processes for metallic materials, based on powder-bed fusion. It can be defined as a process whereby 3D functional parts can be produced by selectively scanning and consolidating a powder bed in a layer-wise manner. SLM provides many advantages over conventional processes such as ability to create complex geometries with internal cavities or features without specific dies or tools, reduced lead time from design to testing, reduced need for assemblies, and joining processes resulting in less production costs (15). We use the selective laser melting at manufacturing of our prosthesis.

A good and lasting connection of the implant with the bone tissue is possible when there are sufficient conditions for the bone to grow into the pores of the material, therefore the use of a porous implant may be helpful in solving this problem Surface morphology is an important factor determining long-term implant stability, especially if bone quality is poor. A porous surface improves mechanical interlocking between the implant biomaterial and the surrounding natural tissue, providing greater mechanical stability at this critical interface (16), our prosthesis had Size of pores shape 900 microns air and 100 microns solid, Volume of pores shape to the whole implant shape is 60% and Weight of pores shape to whole implant body is 38%.

Finite element simulation techniques are being developed to provide mechanical responses and are used without any risks. These techniques provide definition about the load and material, as well as the value of the forces, using computation to investigate the biomechanics of the clavicle as a result of the surgical technique (17). Maximum stress occurs at the middle third of the clavicle length, which is a characteristic of clavicle fractures in normal life. During the dynamic strength analysis, the prosthesis in our study showed similar characteristics and resulted in the same range. The bone substitutes also had similar structural properties and compression strength, indicating that they are suitable for different clinical conditions (18, 19).

Finite element method (FEM) is a highly convenient and effective method to analyze near-real situations with the help of real-time, anticipated boundary and loading conditions, especially for biomedical applications. Computational structural analysis provides many approaches to successfully achieve the target. It is very difficult to analyze and predict exact mechanical behavior of adjacent tissues and implant inside the body (*in-vivo*), during the process of healing in orthopedic applications. Therefore, FEA is very helpful in designing and analyzing any medical device for optimality (20).

Using the FE method in our study was to provide a model allowing detecting the changes in bone characteristics leading to the manifestation of pathological conditions. Therefore, the focus in FE analysis is on creating an optimal FE model.

Validating the results of FEA it is extremely important to check the results given by FEA software (21). This was done by comparison with experimental data, and comparison with other similar computation techniques.

Limitations of this technique are the cost because it is custom made and is therefore more expensive, and, if the patient has any contraindications for CT scans or even surgery, this technique will be limited.

In conclusion, we advocate the use of this novel prosthesis for reconstruction of the lateral half of the clavicle after bone defects due to fracture. The advantages of the new prosthesis are that it replicates the dimensions of the normal bone; the patient could use her shoulder and return to activities of daily living and was satisfied with the aesthetics of the shoulder.

## Data Availability Statement

The original contributions presented in the study are included in the article/supplementary materials, further inquiries can be directed to the corresponding author/s.

## Ethics Statement

Written informed consent was obtained from the relevant individual for the publication of any potentially identifiable images or data included in this article.

## Author Contributions

SA did the finite element analysis and the design of the prosthesis. SMA did the surgery. KA manufacturing the prosthesis. AN and EE-S substantial contributions to the conception and design of the work, drafting the work or revising it for important intellectual content, and final approval of the version to be published. All authors contributed to the article and approved the submitted version.

## Conflict of Interest

The authors declare that the research was conducted in the absence of any commercial or financial relationships that could be construed as a potential conflict of interest.

## Publisher's Note

All claims expressed in this article are solely those of the authors and do not necessarily represent those of their affiliated organizations, or those of the publisher, the editors and the reviewers. Any product that may be evaluated in this article, or claim that may be made by its manufacturer, is not guaranteed or endorsed by the publisher.

## Abbreviations

3D: three-dimensional
CT: computed tomography
DASH: disabilities of the arm, shoulder and hand
DICOM: digital imaging and communication in medicine
FEA: finite element analysis
ROM: range of motion
VAS: visual analog scale.
